# Progress in Psychiatry

**Published:** 1902-05-31

**Authors:** 


### Progress in Psychiatry.

**The Insanities of Puberty and Adolescence.**—Insanity proper is very rare in infancy and childhood, and is thus met with only exceptionally before puberty. The mental diseases or derangements of infancy and childhood comprise idiocy, imbecility (including cretinism), feeblemindedness, the deliria of fever and infectious process, and morbid impulses of dangerous and possibly criminal character. Maniacal excitement is rare, and melancholia is rarer still.[1] The so-called "juvenile" form of general paralysis begins to manifest itself shortly before or after puberty.[2] Children with an hereditary taint of insanity which may manifest itself fully in adult life, often exhibit in childhood a peculiar proneness to "terrors, nightmares, nocturnal delirium, visual hallucinations, and morbid impulses of a dangerous character."[3] The mental disorders peculiar to puberty and adolescence are themselves of various forms. The rapid mental and physical developments of puberty constitute a critical stage so far as the mental and moral equilibrium of the individual is concerned. Physical activities hitherto employed chiefly in sport are now diverted or constrained into the serious channels of work and purpose in life. The unchecked kinæsthetic activities, the exuberance of movement, the overflow of sportive and animal spirits, all of which have hitherto been permitted to issue without control of conscious purpose, are now restrained or modified. In intelligent children great capacity for reading and absorbing literature marks this stage. An undue estimate of individual capacity is begotten, partly because the knowledge thus acquired wants the correcting and disillusioning touches of time and experience, and partly because the vigorous metabolic and nutritive process of growth at this period beget overweening feelings of personal strength and power which again hard experience only can correct. Hence an undue conceit of self and an uncritical self-assurence and pugnacity are common traits of the adolescent period of life, especially in boys. The religious and erotic instincts now begin to unfold themselves with great fervour, as shown by "conversions" and religiosity on the one hand, or by ardent emotions prompting to love and heroism on the other. Creative, artistic, poetic, and constructive talents also show themselves at this stage in boys, and to a less extent also in girls, who have inherited superior intellectual endowments from their parents. On the other hand one or more of these traits may be developed in an abnormal or pathological form or otherwise perverted, giving rise to various kinds of mental and moral aberration. Among the more striking conditions, not, however, amounting to insanity, may be mentioned a tendency to solitude and excessive timidity, or the opposite condition of aggressiveness and forwardness, hooliganism, hypochondriacal depression or maniacal exaltation, religious exaltation or depression with delusions of sinfulness, sexual precocity and excessive amativeness or cruelty and destructiveness, and acts of extraordinary and reckless bravery, or of hysterical romancing and lying to court attention. These, however, are, as a rule, only transitory disturbances, the mere oscillations of a forming character whose various and conflicting elements are seeking equilibrium and harmony of action. Other more serious symptoms may appear and give rise to actual forms of insanity. The latter include five main forms of adolescent insanity,[4] viz., a simple form of adolescent dementia (*dementia præcox simplex*), a delusional form (*d. p. hebephrenica*), a catatonic form (*d.p. catatonica*), a paranoid form (*d. p. paranoides*), and a criminal form (*d. p. criminalis*). The periods of puberty and adolescence for the purposes of this chapter may be respectively taken to include the ages of 15 to 17, and 17 to 21 for the female sex, and 16 to 18, and 18 to 25 for the male sex.

**The Insanity of Puberty.**—As development proceeds and the stage of childhood is passed an increased growth of the forebrain takes place in particular and of the body in general during the period of puberty and adolescence. The boy or girl becomes increasingly subject to social restraint, and in most cases the advent of puberty is attended by some form or other of ceremonial which the pubescent boy or girl has to pass through. Parents and guardians impose laws and conventions of which the child had hitherto remained in ignorance. A new world is gradually opened to view. Meanwhile sexual development and differentiation proceed rapidly, the sexual organs (ovaries, testes, external genitals, and in the female the mammæ) develop rapidly, the heart and vascular organs grow *pari passu*, the pelvis in the female and the larynx in both sexes become enlarged, and increase of stature and of bodily weight are attained. The blind organic instincts of sexual life assert themselves at first in the form of vague impulses towards romance, love, religion, or adventure. These impulses and tendencies may take one form or other, according to circumstances, but what characterises them in common is that there is little or no intellectual basis or motive force behind them. They have a blind organic non-rational potency of their own, and it becomes the office of reason to endow them with an intelligible colouring if possible. But whenever the instinct or impulse becomes strong the wisest maxims of intelligence are forgotten, or at any rate they are ignored. In other words, the inhibitory powers of the higher cortical centres are found insufficient should they come into conflict with the organic instincts and impulses referred to above. Usually a compromise is effected in which reason plays the subordinate part and seeks to devise pleas and justification for the line of conduct pursued. Such is in brief the normal *modus vivendi* of organic instinct versus intelligence during puberty. In cases of insanity at this stage the discrepancy is more strikingly shown. The reasoning powers are wholly in abeyance or profoundly weakened and the clinical manifestations are mainly a grotesque combination of the instincts and impulses proper to the stage of puberty. The symptoms may consist of apathy, mutism, and an apparent arrest of the capacity of attention almost amounting to stupor, or there may be a shallow melancholy varied by outbreaks of liveliness and quixotic demonstrations, or the patient may exhibit a series of histrionic attitudes and religious postures intermingled with occasional silly chatter, the whole being marked by a stupid passive resistance to whatever is required to be done. Delusions of an amatory, persecutory, mystico-religious, and hypochrondriacal character are very prone to develop, while dangerous impulses to suicide, homicide, and other forms of violence may occasionally be manifested. Rapid change of mood is very characteristic of such patients. The operation of an hereditary taint, which is frequently observed in cases of the insanity of pubescence, is shown by the occurrence of rapid improvement, frequent relapses, and the eventual persistence of some degree of permanent mental enfeeblement. If hereditary taint is wanting the more purely affective forms of insanity, such as subacute and acute melancholia or mania or mild hallucinatory mental confusion, are the commoner psychoses of this period of life as compared with the other forms which have been already enunciated under the heading of dementia præcox. *Tics* and choreiform disturbances may be conspicuous features in the minor mental ailments of puberty, as well as an extraordinary capacity for fantastic romancing and lying (*pseudologia fantastica*).[5] Girls are exposed to greater risk of mental disorder than boys during the period of puberty (15 to 17 or 18 years), but the period of adolescence (18 years and after) is fraught with greater risk of mental trouble to the male.

[1] Régis, Mental Medicine, 1895. [2] Alzheimer, Thiry, Clouston, Hirsch, and others. See article by Hirschl in Wien. klin. Wochenschr., Nov. 21, 1901. [3] Régis, loc. cit. [4] According to the observations recorded by Magnan, Morselli, Christian, Régis, and Kraepelin among recent observers. [5] A term aptly coined by Dellbrück to designate this peculiar phase.

